# EMDR in Cancer Patients: A Systematic Review

**DOI:** 10.3389/fpsyg.2020.590204

**Published:** 2021-01-18

**Authors:** Alberto Portigliatti Pomeri, Anna La Salvia, Sara Carletto, Francesco Oliva, Luca Ostacoli

**Affiliations:** ^1^State Police Health Service Department, Ministry of Interior, Rome, Italy; ^2^Department of Oncology, 12 de Octubre University Hospital, Madrid, Spain; ^3^Department of Neuroscience “Rita Levi Montalcini”, University of Torino, Torino, Italy; ^4^Clinical Psychology Unit, University Hospital City of Science and Health, Torino, Italy; ^5^Department of Clinical and Biological Sciences, University of Torino, Orbassano, Italy

**Keywords:** EMDR, cancer, PTSD, psycho-oncology, psychological intervention

## Abstract

**Background:** Psychological distress is common among patients with cancer, with severe consequences on their quality of life. Anxiety and depression are the most common clinical presentation of psychological distress in cancer patients, but in some cases cancer may represent a traumatic event resulting in posttraumatic stress disorder (PTSD). Currently, Eye Movement Desensitization and Reprocessing (EMDR) therapy is considered an evidence-based treatment for PTSD, but recent studies also showed its effectiveness for anxiety and depression. The aim of the present systematic review is to summarize the current literature on the effect of EMDR on cancer-related psychological distress.

**Methods:** A literature search was conducted for peer-reviewed articles about “EMDR” and “cancer patients” in the following electronic databases: PubMed, MEDLINE, Science Direct, Google Scholar, and Cochrane library.

**Results:** Our search identified 7 studies in which EMDR was used with a total of 140 cancer patients. The psychiatric diagnosis was PTSD in 3 studies. Otherwise, the diagnosis concerned the anxious and depressive disorder spectrum. Overall, EMDR treatment schedules used were highly heterogeneous, with a different number of sessions (from 2 to 12) and a different duration of therapy (up to 4 months). However, across all studies analyzed EMDR therapy was judged to be adequate in reducing symptoms of psychological distress in this population.

**Conclusions:** According to the results of our analysis, the level of evidence regarding EMDR efficacy in cancer patients is limited by the scarcity of studies and their low methodological quality. Although better quality research is needed, available data suggest that EMDR could be a promising treatment for psychological distress in patients with cancer.

## Introduction

### Psychological Distress and PTSD in Cancer Patients: Its Epidemiology, Impact on Quality of Life, and Impact on Survival

Facing a diagnosis of cancer, a potentially life-threatening event, is physically and emotionally challenging. Psychological distress is defined as “a set of painful mental and physical symptoms that are associated with normal fluctuations of mood in most people” and, in some cases, it may indicate the beginning of a psychological clinical condition (American Psychology Association). In cancer care, psychological distress has been recognized as the sixth vital sign (National Comprehensive Cancer Network, 2018). Recent analyses across the trajectory of cancer patients have showed that the prevalence of psychological distress ranges between 29 and 59.3% (Zabora et al., 2001; Gao et al., 2010). Cancer patients must face many possible stressors during the course of the disease, such as the diagnosis, the impact of the unfavorable prognosis, the frequent lack of a curative treatment, physical deterioration, and increasing economic burden (Meijer et al., 2013; Baker et al., 2016). Furthermore, cancer patients experience impaired quality of life, and psychological problems have been identified as one of the contributors to reduced quality of life (Marandino et al., 2018). In cancer patients, there is a significant correlation between somatic diseases, functional limitations and psychological distress (Hurria et al., 2009; Given and Given, 2010). The prevalence of psychological distress varies depending on age, gender, type of cancer, illness duration, degree of patient burden, disease progression, prognosis, treatment situation, and other variables (Zabora et al., 2001; Herschbach et al., 2004). A study on ~4,500 patients showed that the prevalence of psychological distress varies between 29 and 43% in patients with the 14 most common cancers, with patients with lung, brain, and pancreatic cancer experiencing the highest levels of psychological distress (Zabora et al., 2001). Another study using a cancer-specific distress questionnaire on ~1,700 patients with 12 different diagnostic subgroups revealed a prevalence of psychological distress ranging between 23.5% in patients with cancer of the upper gastrointestinal tract and 40.9% in patients with breast cancer, with patients with soft tissue tumors and breast cancer having the highest stress scores (Herschbach et al., 2004). Anxiety and depression are considered to be the most common clinical presentation of psychological distress in cancer patients (Cohen, 2014; Weiss Wiesel et al., 2015). However, psychological distress can evolve or be replaced by a more severe and complex scenario represented by posttraumatic stress disorder (PTSD). PTSD is a psychiatric disorder that can occur after experiencing a traumatic event and involves symptoms such as re-experiencing the event itself, the avoidance of trauma-related stimuli, negative alterations in thoughts and feelings, and hyperarousal. The diagnostic criteria for PTSD have been substantially updated in the fifth edition of the American Psychiatric Association's Diagnostic and Statistical Manual of Mental Disorders (DSM-5®)(American Psychiatric Association, 2013), as compared with the fourth edition (DSM-IV-TR®)(American Psychiatric Association, 2000). PTSD now belongs to a new category, called “Trauma- and Stressor-Related Disorders,” and avoidance has been added as one of the required criteria, negative cognitions are highlighted, and traumatic events are not defined by an initial reaction of fear, horror, or helplessness (American Psychiatric Association, 2013). Traditionally, PTSD has been described as a pathological reaction to external traumatic events such as war and natural catastrophes. In recent decades, a growing body of published data supports the existence of PTSD induced by life-threatening experience, such as the complex, uncertain, and somehow treacherous path from the diagnosis of cancer to the subsequent sequences of treatments and their impact on patients' quality of life and survival (Kangas et al., 2002, 2007; Leano et al., 2019).

About 12% of patients present symptoms such as flashbacks, avoiding cancer-related experiences, and increased levels of anxiety approximately 2 months after diagnosis of head and neck cancer (Posluszny et al., 2015; Richardson et al., 2016a). However, a 2013 study at Columbia University revealed that ~25% of breast cancer patients experienced symptoms of PTSD along their therapeutic path (Vin-Raviv et al., 2013). PTSD prevalence rates of 5–17% have been reported in cancer survivor groups (Kornblith et al., 2003; Black and White, 2005). Additionally, roughly 20–29% of cancer caregivers meet the criteria for PTSD (Donnelly et al., 2008; Richardson et al., 2016b). Different aspects of the cancer experience may represent traumatic events triggering cancer-related PTSD, including the shock of the diagnosis, multiple diagnostic tests, the burden of treatments, possible side-effects and medical complications, poor prognosis, continuous monitoring, and fear of recurrence (Ghazali et al., 2013; Cordova et al., 2017; Leano et al., 2019). A recent review article identified childhood trauma, previous PTSD or other psychiatric conditions, low socioeconomic status, young age, advanced stage of disease, short time since treatment, invasive treatment, limited social support, and dissociative symptoms regarding the cancer experience as risk factors for cancer-related PTSD (Cordova et al., 2017). Moreover, higher levels of inflammation and the long-term effect of antiendocrine therapies were found to be correlated with PTSD in patients with breast cancer (Brown et al., 2020). It should also be mentioned that there is usually a large time gap between PTSD symptoms and the initiation of its treatment (Kadan-Lottick et al., 2005; Kaidar-Person et al., 2017). However, when symptoms of trauma become more pervasive and fail to fade over time, clinical intervention might be necessary.

### EMDR: Features, Indications, Evidence

EMDR is an eclectic model of psychotherapy that was developed in the 1980s by the psychologist Francine Shapiro (1989), who originally described a standard intervention protocol that centered on working on traumatic memories and the stress symptoms associated with them. The EMDR approach is guided by the Adaptive Information Processing model (AIP; Shapiro, 2001), which posits that most psychopathology is caused by unprocessed disturbing memories. A central part of the EMDR procedure consists of the patient recalling traumatic memories while simultaneously making horizontal eye movements or receiving other kinds of bilateral stimulation (BLS), such as alternating left and right beeps or tapping. BLS is thought to elicit a sort of accelerated information processing that desensitizes the most disturbing aspects of traumatic memories. This approach aims to reprocess these memories and include them within the patient's normalized biographical memories (Amano and Toichi, 2016).

The core component of EMDR consists of keeping the patient's attention focused on two different things, the traumatic memory and the rhythmic BLS. This state of attention is fundamental since it can induce certain physiological conditions that, in turn, activate information processing. EMDR therapy is an eight-phase treatment, characterized as follows: Phase 1: history-taking, to identify traumatic memories; Phase 2: stabilization, to prepare the patients for treatment by stabilizing and increasing access to positive resources; Phases 3-6: a target memory is identified and processed using EMDR therapy procedures (i.e., eye movements or other BLS); Phase 7: closure, the patient is reminded to use the self-calming activities that were mastered in Phase 2; Phase 8: reevaluation, which occurs at the beginning of the subsequent session to check whether achieved results are maintained or need further reprocessing. A full description of the sequence of treatment can be found in Shapiro (2001).

Recently, a specific EMDR therapy protocol for patients with cancer has been developed (Faretta and Borsato, 2016). The EMDR intervention in psycho-oncology is focalized on disease-related memories and on present difficulties related to the different stages of the illness. Therefore, the main difference in this protocol is represented by the target selection (Phase 3), as “priority is given to targets of the present and/or recent past that are connected with the experience of illness” (Faretta and Borsato, 2016).

The main hypotheses related to the EMDR mechanism of action suggested possible explanations of the effect of eye movements such as the increasing cerebral hemispheres connectivity through neuronal activation (Christman et al., 2003; Propper et al., 2007; Parker et al., 2008); the activation of neurological mechanisms similar to those of the REM and SWS sleep phases (Stickgold, 2002; Pagani et al., 2017); “to exhaust” the working memory altering the quality of storage of traumatic memories, reducing the intensity of episodic memories and, with this, the symptoms of PTSD (Maxfield, 2008; van Schie et al., 2016; van Veen et al., 2019).

Official clinical guidelines support EMDR as an intervention for adults with PTSD (World Health Organization, 2013; Courtois et al., 2017; National Institute for Health Care Excellence, 2018; International Society of Traumatic Stress Studies, 2019). A recent network meta-analysis has concluded that EMDR, along with trauma-focused cognitive behavior therapy (TF-CBT), appears to be most effective at reducing symptoms and improving remission rates in adults with PTSD, even in the long term (Mavranezouli et al., 2020). In the recent years, the application of EMDR beyond PTSD has expanded rapidly and research has underlined its efficacy to successfully treat other psychological disorders (Valiente-Gómez et al., 2017). Preliminary evidences suggest that EMDR can be a useful treatment for both depression (Carletto et al., 2017; Malandrone et al., 2019) and anxiety disorders (Faretta and Dal Farra, 2019; Yunitri et al., 2020). Finally, a previous review has shown that trauma-focused interventions are effective in reducing PTSD symptoms in patients with cancer (Dimitrov et al., 2019). Therefore, the aim of the present systematic review is to summarize the current literature on the effect of EMDR on cancer-related psychological distress.

### Aims of the Study

This review aims to interrogate the literature regarding the role and magnitude of the impact of EMDR in cancer patients. By integrating evidence relating to all aspects of the EMDR approach in cancer patients, this review aims to provide a comprehensive overview of the evidence base to inform the development of EMDR interventions in this context.

## Materials and Methods

### Design

An integrative review was undertaken, according to the systematic review methods advocated for by the Cochrane Collaboration, the Scottish Intercollegiate Guideline Network (SIGN) (Scottish Intercollegiate Guidelines Network, 2008), and AMSTAR standards for reporting systematic reviews. The review and reporting of results were guided by the PRISMA checklist. Ethical approval was not required. The present review was registered in the PROSPERO system, ID CRD42020187658.

### Search Strategy

**Figure 1:**
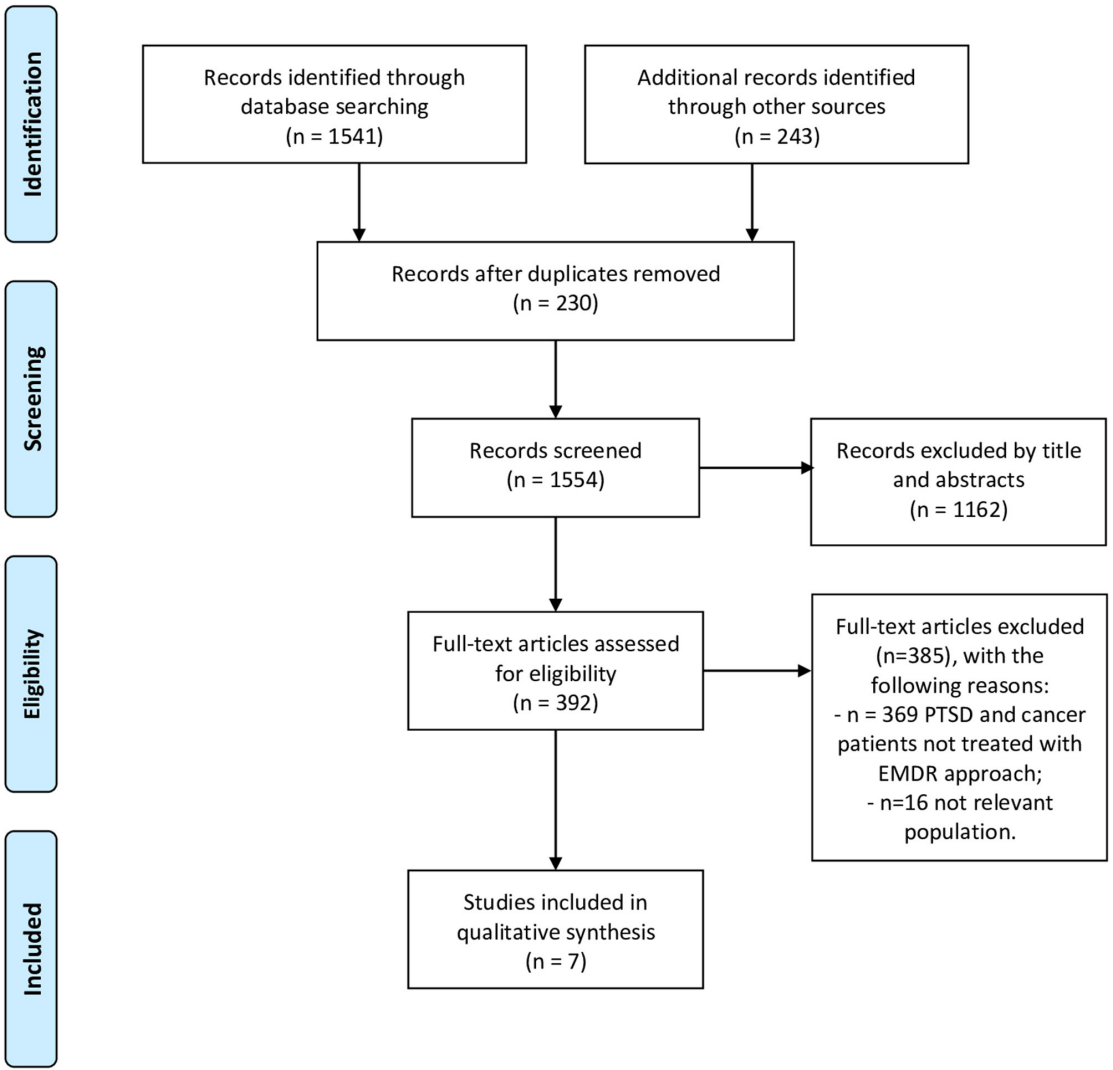
PRISMA flow diagram.

Literature search: Peer-reviewed research articles regarding EMDR and cancer patients were identified through searches in the following electronic databases: PubMed, MEDLINE, Science Direct, Google Scholar, and Cochrane library (Figure 1, according to PRISMA 2009 Flow Diagram). The combinations of keywords relating to the proposed objectives were: EMDR and cancer, and psychological distress and PTSD in cancer patients. Reference lists of included articles were also searched.

The following question and the PICO (i.e., Population Intervention Comparison Outcome) elements form the basis for the review:

Is EMDR an efficacious and safe option for the treatment of psychological distress in cancer patients?

The question categorized into PICO elements:

P: Cancer patients

I: Eye movement desensitization and reprocessing (EMDR)

C: Alternative to EMDR (watch and waiting, psychiatric medications, other psychological interventions)

O: effects on psychological distress.

### Selection Criteria

Studies were included if the following pre-specified criteria were met:

- Published papers including quantitative data about the effect of EMDR in patients with any type of cancer.
- Only articles published in English were considered.

Papers were excluded if they did not focus, at least in part, on EMDR in relation to patients with cancer. Moreover, qualitative reports were excluded.

### Study Selection and Appraisal

Two authors (APP and ALS) independently reviewed titles and abstracts and excluded irrelevant articles and duplicates, and then retrieved the full texts for all remaining articles. Disagreements were resolved by discussion between the two review authors.

The internal validity of the randomized controlled trials was assessed by using Version 2 of the Cochrane risk-of-bias tool for randomized trials (RoB2) (Sterne et al., 2019). The quality of controlled studies was assessed using the MINORS Scale (Slim et al., 2003; Zeng et al., 2015). The Joanna Briggs Institute (JBI) Case Reports Critical Appraisal Tool (Moola et al., 2017) was used for case reports. The methodological quality was assessed by two independent reviewers (SC and FO) and any disagreements were solved through group discussion.

### Data Extraction

The data were extracted independently by two reviewers (APP and ALS). Extracted data covered: study design, information about sample characteristics and type of psychiatric disorder, type of cancer, type of intervention and comparison, type of assessment tools used to assess psychological distress, and principal results reported by the authors.

## Results

Our search identified seven studies in which the EMDR technique was used on patients with an oncological diagnosis (Capezzani et al., 2013; Jarero et al., 2015; Faretta et al., 2016; Szpringer et al., 2018; Borji et al., 2019; Carletto et al., 2019; Dinapoli et al., 2019). Each of the 7 papers were independently read and assessed by APP and ALS for relevance. The main characteristics of the studies analyzed are reported in Table 1.

**Table 1:** Characteristics of the included studies.

**References**	**Design**	**Comparison (*N* of patients)**	**Gender/Age**	**Diagnosis**	**Type of tumor**	**EMDR Treatment schedule**	**Psychometric tests**	**Results**
Capezzani et al. (2013)	RCT	EMDR (*n* = 11, follow-up stage; *n* = 10, active cancer treatment) CBT (*n* = 10, follow-up stage)	28 female, 3 male/EMDR = mean age 53.40 (active treatment); mean age 50.82 (follow-up stage); CBT = mean age 52.70	PTSD	Mixed cancer diagnosis	8 weekly sessions	CAPS, IES-R, BDI-II, STAY-Y, QPF-R	Reduction of PTSD symptoms and PTSD diagnosis after EMDR interventon; EMDR>CBT.
Jarero et al. (2015)	Pre-post	EMDR (*n* = 17, active cancer treatment; *n* = 7, follow-up stage)	All female/mean age 54.16	PTSD	Mixed cancer diagnosis	3 consecutive days, twice daily sessions	SPRINT	Reduction of PTSD after EMDR intervention, maintained over time.
Faretta et al. (2016)	Controlled	EMDR (*n* = 31, active cancer treatment and follow-up stage, not specified) CBT (*n* = 26, active treatment and follow-up stage, not specified)	45 female, 12 male/EMDR = mean age 51.45; CBT = mean age 53.28	Anxiety and depression disorder spectrum	Mixed cancer diagnosis	12 sessions	SCL-90-R, COPE, DTS	Reduction of psychological symptoms after EMDR intervention; EMDR>CBT.
Szpringer et al. (2018)	Controlled	EMDR (*n* = 18, active cancer treatment) Control group (*n* = 19, active treatment)	All female/ EMDR = mean age 63.00; Control group = mean age 65.50	Anxiety and depression disorder spectrum	Glioblastoma multiforme	10–12 sessions during 4 months	HADS, SOC-29	Decrease in anxiety, depression and anger and improve of improve sense of coherence after EMDR intervention; EMDR>control group.
Borji et al. (2019)	RCT	EMDR (*n* = 30, active cancer treatment) Control group (*n* = 30, active cancer treatment)	24 female, 36 male/ EMDR = mean age 70.03; Control group = mean age 68.33	Not specified	Gastrointestinal cancer	2 sessions at home's patient	PSS	Reduction of perceived stress after EMDR intervention
Carletto et al. (2019)	Controlled	EMDR (*n* = 15, active cancer treatment) Control group (*n* = 15, active cancer treatment)	All female/ EMDR = mean age 55.47; Control group = mean age 48.40	PTSD	Breast cancer	10 sessions during 3-4 months	CAPS, IES-R, BDI-II, STAY-Y	Reduction of PTSD symptoms, PTSD diagnosis and depressive symptoms after EMDR intervention; EMDR>TAU.
Dinapoli et al. (2019)	Case study	EMDR (*n* = 1, active cancer treatment)	Male/73 years old	Intolerable anxiety	Head and neck cancer	3 sessions during 2 weeks	HADS, VAS	Reduction of anxiety symptoms after EMDR intervention

*CBT, cognitive behavioral therapy; PTSD, posttraumatic stress disorder; QPF-R, psychophysiological questionnaire-brief version; STAI-Y, state-trait anxiety inventory; BDI-II, Beck depression inventory-II; IES-R, impact of event scale-revised; CAPS, clinician-administered PTSD Scale; SPRINT, short PTSD rating interview; SCL-90-R, symptom checklist-90-r; COPE, cope inventory; DTS, Davidson trauma scale; HADS, hospital anxiety and depression scale; SOC-29, sense of coherence scale; PSS, perceived stress scale; VAS, visual analog scale*.

Only three studies used a control group, and in two studies the EMDR technique was compared to CBT. Only two studies utilized randomized clinical trials (Capezzani et al., 2013; Borji et al., 2019). One study was a case report of a man with a squamous cell carcinoma of the glottic larynx.

Altogether, the seven studies included a total of 140 patients. Patients' gender and age were not always specified in the description of the population. Patients were diagnosed with PTSD in three out of seven studies. Otherwise, the diagnosis concerned the anxiety-depression spectrum. The oncological diagnosis in terms of tumor type of the patients included in these analysis was extremely heterogeneous. In three studies, the criteria of a unique tumor diagnosis was not followed. In the remaining research, patients were suffering from breast cancer (one study), glioblastoma multiforme (one), gastrointestinal cancer (one), and head and neck cancer (one).

Overall, the treatment schedule used by the researchers to administer EMDR therapy was considerably heterogeneous. Different numbers of sessions (from two to twelve) of EMDR and different durations of therapy (up to 4 months) were applied in the studies. The psychometric tests were the most varied: two out of three studies with PSTD patients employed scales expressively used to assess PTSD symptoms like IES-R (Impact of Event Scale-Revised) and CAPS (Clinician-Administered PTSD Scale), whereas the third study used the SPRINT scale (Short PTSD Rating Interview). In the other studies, scales to evaluate depressive and anxiety symptoms like HADS (Hospital Anxiety and Depression Scale), BDI-II (Beck Depression Inventory-II), and STAY-Y (State-Trait Anxiety Inventory) were applied. In one study, the sense of coherence with the SOC-29 (Sense of Coherence Scale) was evaluated and in another the PSS (Perceived Stress Scale) was used.

Finally, the efficacy of EMDR therapy on these patients was adequate in all trials in reducing symtoms and most beneficial when compared to CBT in two studies.

### Quality Appraisal of Studies

Risk of bias and the methodological quality of each study is reported in Supplementary Material. The two randomized controlled trials were assessed with RoB2. Several methodological weaknesses were found. The major issues identified were related to the randomization process and allocation. All controlled studies showed a high risk of bias, not including the prospective collection of data and calculation of sample size. Endpoints were appropriate to the aim in only two studies, and none guaranteed an unbiased assessment of the study endpoint applying blinding of post-treatment evaluators.

## Discussion

Recent data have suggested that EMDR therapy can be considered a promising treatment for those affected by cancer in providing support to patients, their families, and professional caregivers (Faretta and Borsato, 2016). This study represent the first attempt to summarize available findings on the effect of EMDR for reducing psychological distress in cancer patients. Currently, EMDR is considered an evidence-based treatment only for PTSD (World Health Organization, 2013; National Institute for Health Care Excellence, 2018). Two recent systematic reviews have suggested that EMDR could be an add-on treatment in chronic pain conditions (Tesarz et al., 2014; Valiente-Gómez et al., 2017).

Psychological distress should be considered a common finding among cancer patients and survivors, with a considerable impact on their quality of life (Abbey et al., 2015; Swartzman et al., 2017; Harms et al., 2019). Previous evidence suggests that cancer patients do not form a homogenous group when it comes to levels of psychological distress; for example, subjective distress has been shown to differ according to the specific type of cancer, such as prostate and breast cancer, amongst others (Brintzenhofe-Szoc et al., 2009; Admiraal et al., 2013). Other studies have instead reported that psychological distress was related to gender and age but not to cancer type (Lavelle et al., 2017).

Psychological treatments may be beneficial in alleviating distress and improving the quality of life of cancer patients (Murray, 2016). Consistent data have demonstrated how TF-CBT, stress management group cognitive-behavioral therapy, and EMDR are able to improve PTSD symptoms more than waiting-list or as usual treatments (Haerizadeh et al., 2020). A meta-analysis including 114 randomized controlled trials showed that TF-CBT and EMDR are the psychological therapies with the strongest evidence of effect for the treatment of PTSD (Lewis et al., 2020). Furthermore, a recent increase in the number of randomized controlled trials of psychological therapies for PTSD resulted in a more confident recommendation of TF-CBT and EMDR as first-line treatments. However, the expert recommendation regarding the treatment of cancer-related PTSD is that it should be approached with caution and be informed by existing evidence-based approaches for traumatic stress (Kangas, 2013; Kangas et al., 2013; Cordova et al., 2017). Although stressing the limits due to the low number of studies included, a recent review has provided evidence supporting the use of trauma-focused interventions (i.e., EMDR and CBT) in treating cancer-related PTSD symptoms (Dimitrov et al., 2019).

Previous research has hypothesized that the functional effects of EMDR therapy can represent an ideal treatment option in the setting of patients with cancer and consequent PTSD (Carletto and Pagani, 2016). The authors suggest that EMDR has a proven ability to normalize the dysfunction of limbic areas involved in PTSD and depression, and might relieve patients from the psychological burden associated with cancer.

Our review found that there were relatively few studies investigating the use of EMDR psychotherapy for cancer patients. We identified only seven articles that fulfill our inclusion criteria. There were two notable findings from our review of eligible trials: (1) all of the included trials reported that EMDR interventions were associated with significantly lower psychological distress and post-treatment mood disorder spectrum symptoms and, (2) EMDR proved to be superior to CBT in two of the trials analyzed.

However, there are several limitations to be considered. Our retrospective collection of data included a limited number of highly heterogeneous studies concerning cancer patients experiencing psychological distress symptoms or mood disorder diagnoses treated with EMDR therapy. Unfortunately, the cancer population considered altogether is extremely variegated in terms of type of cancer as well as disease stage, treatments received for the oncological disease, and socio-demographic variables (i.e., age and gender). An important limitation is also related to the several methodological flaws identified in the included studies; almost all were judged as having a high risk of bias. Moreover, the included studies tested dissimilar EMDR interventions in terms of both number and length of session. Therefore, larger and more robust evaluations are needed to draw firm conclusions regarding the efficacy of EMDR in this population.

The measures used in the reported studies appeared to be heterogeneous but appropriate. The CAPS, a clinical semi-structured interview based on the DSM-IV-TR, is the gold standard to assess PTSD (Blake et al., 1995; Weathers et al., 2001). It is used in two of the three studies related to PTSD. In Jarero et al. (2015), the researchers administered the SPRINT, an eight-item self-report measure that assesses the core symptoms of PTSD and social functional impairment. This scale has demonstrated solid psychometric properties (Connor and Davidson, 2001). As regards the other scales aimed at measuring the symptoms of anxiety, depression, and stress, validated and common scales used in internal medical contexts, in particular the HADS and the SCL-90, were administered in the considered studies (Wang et al., 2017; Annunziata et al., 2020). One of the limits of our review was the exclusion of non-English articles.

This review reports a summary of the published experiences of the application of EMDR psychotherapy in patients with cancer.

At present, research in this area is still in an early phase. Available data suggest that EMDR could be considered a potentially effective treatment for psychological distress in cancer patients. However, due to the high heterogeneity and several methodological limitations of the studies conducted so far, further dedicated clinical trials are highly encouraged before treatment recommendations can be made in this setting.

## Data Availability Statement

The original contributions presented in the study are included in the article/Supplementary Material, further inquiries can be directed to the corresponding author.

## Author Contributions

APP, ALS, and LO were responsible for the conception and the design of the study. APP, ALS, and FO were responsible for the acquisition of data. LO and SC contributed to the interpretation of data. APP, ALS, and SC wrote the article, which was critically revised by all the other authors. All authors have approved the final version of the manuscript.

## Conflict of Interest

LO is an EMDR supervisor. SC and LO have been invited speakers at national and international EMDR conferences. The remaining authors declare that the research was conducted in the absence of any commercial or financial relationships that could be construed as a potential conflict of interest.
