# Supplementary material for: EMDR in Cancer Patients: A Systematic Review
Source: Front Psychol. 2021 Jan 18;11:590204. doi: 10.3389/fpsyg.2020.590204 (PMC7847844; doi:10.3389/fpsyg.2020.590204)
Supplement: Supplementary file 1 [file Table_1.DOCX]

# **SUPPLEMENTARY MATERIAL.** **RISK OF BIAS AND METHODOLOGICAL QUALITY OF INCLUDED STUDIES.**

# **S1. Risk of bias of RCTs - Cochrane risk-of-bias tool for randomized trials (RoB2)**

| Author, year | Randomization process | Deviations from intended interventions | Missing outcome data | Measurement of the outcome | Selection of the reported result | RoB2 Overall Bias |
| --- | --- | --- | --- | --- | --- | --- |
| Capezzani et al., 2013 | Some concerns | Low | Low | Some concerns | Some concerns | Some concerns |
| Borji et al., 2019 | High | High | Low | Some concerns | Some concerns | High |

# **S2. Risk of bias of controlled studies - MINORS Scale**

| **Author, year** | **Item 1** | **Item 2** | **Item 3** | **Item 4** | **Item 5** | **Item 6** | **Item 7** | **Item 8** | **Item 9** | **Item 10** | **Item 11** | **Item 12** | **MINORS total Score** | **MINORS  overall risk of bias** |
| --- | --- | --- | --- | --- | --- | --- | --- | --- | --- | --- | --- | --- | --- | --- |
| Faretta et al., 2016 | 2 | 2 | 1 | 1 | 1 | 1 | 2 | 0 | 2 | 2 | 1 | 2 | 17 | High |
| Jarero et al., 2015 | 2 | 1 | 1 | 2 | 1 | 2 | 1 | 0 | - | - | - | - | 10 | High |
| Szpringer et al., 2018 | 2 | 0 | 1 | 2 | 0 | 1 | 2 | 0 | 1 | 2 | 1 | 1 | 13 | High |
| Carletto et al., 2019 | 2 | 2 | 1 | 1 | 2 | 1 | 2 | 0 | 1 | 2 | 1 | 2 | 17 | High |

MINORS Scale items: 1) A clearly stated aim; 2) Inclusion of consecutive patients; 3) Prospective collection of data; 4) Endpoints appropriate to the aim of the study; 5) Unbiased assessment of the study endpoint; 6) Follow-up period appropriate to the aim of the study; 7) Loss to follow up less than 5%; 8) Prospective calculation of the study size; *Additional criteria in the case of comparative study*: 9) An adequate control group; 10) Contemporary groups; 11) Baseline equivalence of groups; 12) Adequate statistical analyses. The items are scored 0 (not reported), 1 (reported but inadequate) or 2 (reported and adequate). MINORS overall risk of bias: 'Low' risk of bias was considered when studies fulfilling all MINORS criteria with a score =2. 'Some concerns' was rated when studies were judged to raise some concerns in at least one domain, but not to be at 'High' risk of bias for any domain. 'High' risk of bias was applied to all other studies.

# **S3. Methodological quality of case report - JBI Critical Appraisal Checklist for Case Reports**

| **Author, year** | **Item 1** | **Item 2** | **Item 3** | **Item 4** | **Item 5** | **Item 6** | **Item 7** | **Item 8** | **JBI overall appraisal** |
| --- | --- | --- | --- | --- | --- | --- | --- | --- | --- |
| Dinapoli et al., 2019 | Yes | No | Yes | Yes | Yes | Yes | No | Yes | Include |

JBI Critical Appraisal Checklist for Case Reports items: 1) Were patient’s demographic characteristics clearly described?; 2) Was the patient’s history clearly described and presented as a timeline?; 3) Was the current clinical condition of the patient on presentation clearly described?; 4) Were diagnostic tests or assessment methods and the results clearly described?; 5) Was the intervention(s) or treatment procedure(s) clearly described?; 6) Was the post-intervention clinical condition clearly described?; 7) Were adverse events (harms) or unanticipated events identified and described?; 8) Does the case report provide takeaway lessons? Answers: Yes, No, Unclear or Not/Applicable.
